# In vitro reconstitution and characterisation of the oxidative d-xylose pathway for production of organic acids and alcohols

**DOI:** 10.1186/s13568-019-0768-7

**Published:** 2019-04-11

**Authors:** Harry Boer, Martina Andberg, Robert Pylkkänen, Hannu Maaheimo, Anu Koivula

**Affiliations:** VTT Technical Research Centre of Finland Ltd., P.O. Box 1000, 02044 VTT Espoo, Finland

**Keywords:** Dahms pathway, In vitro enzyme pathway, Glycolate, Ethylene glycol, Lactate, Lactonase

## Abstract

The oxidative d-xylose pathway, i.e. Dahms pathway, can be utilised to produce from cheap biomass raw material useful chemical intermediates. In vitro metabolic pathways offer a fast way to study the rate-limiting steps and find the most suitable enzymes for each reaction. We have constructed here in vitro multi-enzyme cascades leading from d-xylose or d-xylonolactone to ethylene glycol, glycolic acid and lactic acid, and use simple spectrophotometric assays for the read-out of the efficiency of these pathways. Based on our earlier results, we focussed particularly on the less studied xylonolactone ring opening (hydrolysis) reaction. The bacterial *Caulobacter crescentus* lactonase (*Cc* XylC), was shown to be a metal-dependent enzyme clearly improving the formation of d-xylonic acid at pH range from 6 to 8. The following dehydration reaction by the ILVD/EDD family d-xylonate dehydratase is a rate-limiting step in the pathway, and an effort was made to screen for novel enolase family d-xylonate dehydratases, however, no suitable replacing enzymes were found for this reaction. Concerning the oxidation of glycolaldehyde to glycolic acid, several enzyme candidates were also tested. Both *Escherichia coli* aldehyde dehydrogenase (*Ec* AldA) and *Azospirillum brasilense* α-ketoglutarate semialdehyde dehydrogenase (*Ab* AraE) proved to be suitable enzymes for this reaction.

**Electronic supplementary material:**

The online version of this article (10.1186/s13568-019-0768-7) contains supplementary material, which is available to authorized users.

## Introduction

The production of chemical building blocks and fuels from biomass is a promising option to replace the fossil raw material sources with renewable alternatives. Biocatalysts, i.e. enzymes and microbes, offer a sustainable way to produce chemicals starting from lignocellulose-based sugars. Microbes have evolved to utilise both pentose and hexose sugars, and their multi-enzyme pathways can be applied for production of compounds found in natural metabolism. Furthermore, integration of new enzymes to create synthetic pathways enables the extension towards completely novel chemicals (reviewed in Lee et al. 2011, 2019).

We have been interested to study utilisation of the pentose sugars d-xylose and l-arabinose, abundant especially in grasses, agricultural crops and hardwoods waste material, for sustainable production of fuels and chemicals. The work has focussed particularly in studying and utilising the oxidative microbial pentose pathway, which was first observed in *Pseudomonas fragi* (Weimberg 1961). We have cloned, expressed and characterised several microbial enzymes involved in these pathways and also determined 3D structures of the enzymes (Toivari et al. 2012; Aro-Kärkkäinen et al. 2014; Andberg et al. 2016; Rahman et al. 2017, 2018). Furthermore, the microbial enzymes have also been used to engineer yeasts to produce, e.g. d-xylonic and l-arabonic acid (Nygård et al. 2011; Toivari et al. 2012, 2013; Aro-Kärkkäinen et al. 2014) which have potential applications as chelator, dispersant, clarifying agent, antibiotic, health enhancer, polyamide or hydrogel modifier, or as l-1,2,4-butanetriol precursor used for manufacturing the propellant 1,2,4-butanetriol trinitrate (Niu et al. 2003; Cao et al. 2015).

**Fig. 1:**
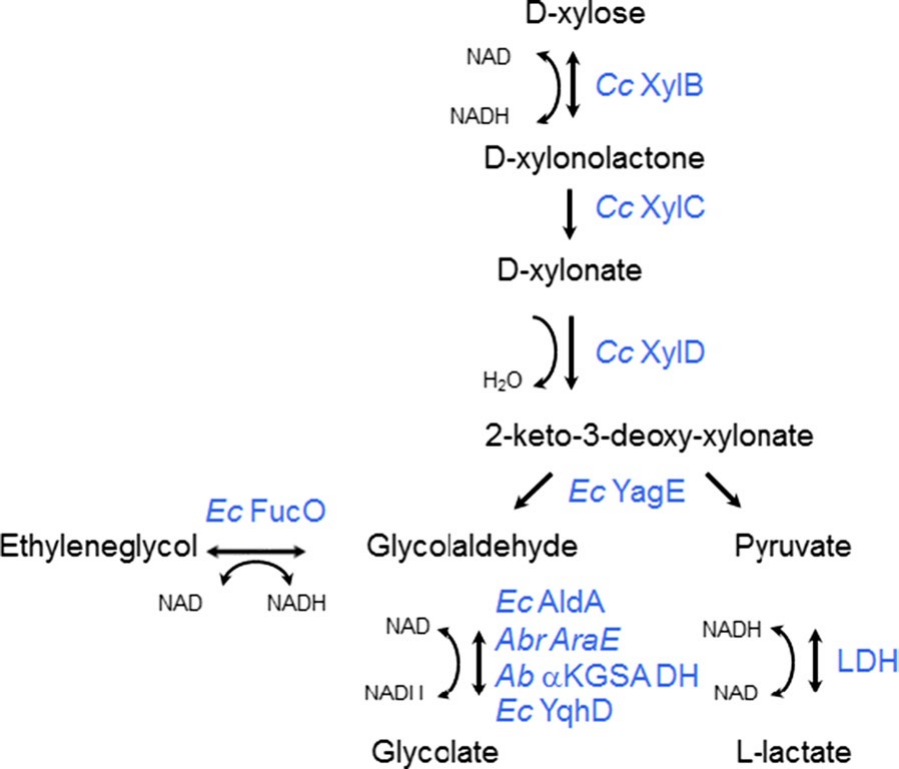
Oxidative d-xylose pathway enzymes including the additional enzymatic steps to produce glycolic acid, ethylene glycol or lactic acid. The enzyme abbreviations are summarized in Table 1

The microbial, non-phosphorylative oxidative pathways contains similar enzymatic steps for both d-xylose and l-arabinose sugars. Concerning the xylose pathway, d-xylose is first oxidized to d-xylonolactone by a d-xylose dehydrogenase, followed by a lactonase to hydrolyze the lactone to d-xylonate. A xylonate dehydratase removes then a water molecule from d-xylonate, resulting in 2-keto-3-deoxy-xylonate, which is a branch point for two different pathways. In the Weimberg pathway (Weimberg 1961), a second hydratase reaction leads to formation of the α-ketoglutarate semialdehyde, which is subsequently oxidized to the tricarboxylic acid cycle intermediate α-ketoglutarate. In the Dahms pathway (Dahms 1974), the 2-keto-3-deoxy-xylonate is split by an aldolase to pyruvate and glycolaldehyde (see also Fig. 1). These two pathways provide possibility for the biosynthesis of a variety of chemicals starting from pentose sugars. As shown by us and other groups, the Dahms pathway can be used, besides xylonic acid (Toivari et al. 2012), also for production of ethylene glycol (Liu et al. 2013; Salusjärvi et al. 2019), glycolic acid (Salusjärvi et al. 2017, 2019), lactic acid (Penttilä et al. 2014), and 1,4-butanediol (Liao and Yan 2011; Tai et al. 2016).

Lactic acid is one of the most known products of the biotech industry used for making polylactic acid (PLA). Lactic acid can be derived from pyruvate (one of the end-products from Dahms pathway) by lactate dehydrogenase (Fig. 1). Ethylene glycol is traditionally produced from ethylene, a main product of the petrochemical industry (Harris 2013). Ethylene glycol is used in polyethylene terephthalate (PET) plastics, and also as an anti-freezing agent and coolant (Baudot and Odagescu 2004). An ethylene glycol producing *E. coli* strain has been constructed by co-expressing the xylose dehydrogenase, a xylonate dehydratase, a 2-dehydro-3-deoxy-d-pentonate aldolase, and finally an aldehyde reductase (Liu et al. 2013). The latter enzyme reduces the glycolaldehyde, derived from the Dahms pathway, to ethylene glycol. Furthermore, glycolic acid, the smallest α-hydroxy acid containing both an alcohol and a carboxyl group, is used in cosmetics, water treatment and industrial and household cleaning applications, as well as in polymers (Babilas et al. 2012). Glycolate production has been demonstrated in engineered microbial hosts, by utilising the glyoxylate cycle in *E. coli* (Martin et al. 2013), *Saccharomyces cerevisiae* (Koivistoinen et al. 2013), and *Kluyveromyces lactis* (Koivistoinen et al. 2013). In addition Salusjärvi et al. 2017, demonstrated recently the production of ethylene glycol and glycolate in *S. cerevisiae* using an oxidative pathway starting from d-xylose. Here, the glycolate was obtained from glycolaldehyde using *E. coli* aldehyde dehydrogenase (*Ec* AldA) (The enzyme abbreviations are summarized in Table 1).

**Table 1:** The table below gives an overview of the enzymes used in this study

Enzyme	NCBI or Uniprot identifier	Function	EC number	Microbial origin	Purification tag included	Expression host (used in this work)
*Cc* XylB	GI: 614102613	Dehydrogenase	1.1.1.175	*Caulobacter crescentus*	Strep-II + TEV_N	*S. cerevisiae*
*Cc* XylC	CC_0820 GI: 16125073	Lactonase	3.1.1.68	*C. crescentus*	No tag	*E. coli*
*Cc* XylD	GI: 1043567249	Dehydratase	4.2.1.82	*C. crescentus*	Strep-II_N	*E. coli*
*Ec* YagE	b0268 GI: 357529065	Aldolase	4.1.2.20	*E. coli*	Strep-II_N	*E. coli*
*Ec* AldA	b1415 GI: 113602	Oxidoreductase	1.2.1.22	*E. coli*	His_6_-tag_N	*E. coli*
*Ab* AraE	GI: 40339944	Oxidoreductase	1.2.1.24 1.2.1.26	*Azospirillum brasieliense*	His_6_-tag_N	*E. coli*
*Ab* α-KGSA DH	GI: 81613403	Dehydrogenase	1.2.1.26	*Acinetobacter baylyii* ADP1	His_6_-tag_N	*E. coli*
*Ec* YqhD	b3011 GI: 301015215	Oxidoreductase	1.1.1.-	*E. coli*	His_6_-tag_N	*E. coli*
LDH	Commercial	Dehydrogenase	1.1.1.27	Rabbit muscle	–	–
*Ec* FucO	b2799 GI: 357528800	Oxidoreductase	1.1.1.77	*E. coli*	His_6_-tag_C	*E. coli*
*Hm* XylD	dgoA4, GI: 55230170	Dehydratase	4.2.1.82	*Haloarcula marismortui*	Strep-II_N	*E. coli*
*Hv* XylD	GI: 292493977	Dehydratase	4.2.1.82	*Haloferax volcanii*	Strep-II_N	*E. coli*
*Se* GluDHT	GI: 667467043	Dehydratase	4.2.1.-	*Salmonella enterocolica*	Strep-II_N	*E. coli*
*Rx* MR/MLE	GI: 123368307	Dehydratase	4.2.1.-	*Rubrobacter xylanophilus*	His_6_-tag_C	*E. coli*
*Pa* GalDHT	UniProt: D4GJ14	Dehydratase	4.2.1.-	Pantoea ananatis	His_6_-tag_N	*E. coli*

The NCBI/Uniprot identifier, function, EC number, microbial origin, purification tag and expression host are specified

In this article, we present the in vitro reconstitution of the oxidative d-xylose pathway for production of the biotechnologically important products ethylene glycol, glycolic acid and lactic acid (Fig. 1). We wanted to study different enzymes for the selected reactions within the pathways and also to characterise some less well known enzymes, such as the lactonase. The data presented provides insights on the action of individual enzymes and their behaviour as part of the pathway, and is relevant for the development of in vitro multi-enzyme cascade reactions or optimization of engineered microbial strains used for the biosynthesis of the platform chemicals.

## Materials and methods

### Chemicals and enzymes

Glycolaldehyde dimer, d-xylono-1,4-lactone, dl-lactaldehyde, d-xylonic acid lithium salt were purchased from Sigma-Aldrich UK. Zinc chloride was purchased from Merck, Germany. Mammalian hydroxyacid oxidase 1 (or glycolate oxidase, HAO1) enzyme was obtained from MyBioSource USA and LDH (lactate dehydrogenase) from rabbit muscle was purchased from Sigma-Aldrich. 10-Acetyl-3,7-dihydroxyphenoxazine (or Amplex Red) was purchased from Sigma-Aldrich. All other substrates were purchased from Sigma-Aldrich in high purity grade. The *E. coli* BL21(DE3) strain was used for the pBAT4 (Peränen et al. 1996) based cytoplasmic expression vectors. LB growth media was prepared according to Sambrook and Russel (2001). Glucose release medium EnPresso B was obtained from Bio-Silta Ltd. Ampicillin resistance (100 μg ml^−1^) was used for the selection of all plasmids. d-xylono-1,4-lactone was analysed by ^1^H-NMR spectroscopy to verify it has not spontaneously opened to d-xylonic acid.

### Cloning of the pathway genes

The d-xylonolactonase encoding gene from *C. crescentus* (Cc *xylC*, CC_0820, Accession: NP_419637, GI: 16125073, NCBI), the aldehyde dehydrogenase encoding gene from *E. coli* (Ec *aldA*, b1415, Accession: P25553.2, GI: 113602, NCBI), and *E. coli* 2-dehydro-3-deoxy-d-pentonate aldolase encoding gene (*Ec yagE*, b0268, Accession: P75682.2, GI: 357529065, NCBI) were purchased as synthetic genes, and codon optimized for *E. coli* (GenScript, China) in the pBAT4 vector (Peränen et al. 1996). The genes encoding *Azospirillum brasiliense* α-ketoglutarate semialdehyde dehydrogenase (*Ab araE*, Accession: Q1JUP4.1, GI: 40339944, NCBI) *E. coli* 1,3-propanediol oxidoreductase (*Ec yqhD,* Accession: ADK47404.1, GI: 301015215) and *E. coli* 1,2-propanediol oxidoreductase (*Ec fucO*, Accession: P0A9S1.2, GI: 357528800, NCBI) were obtained as synthetic genes codon optimized for *E. coli* (GenArt, Thermo Fisher, Germany) and inserted into the linearized (*Nco*I and *Xho*I digestion) pBAT4 vector (Peränen et al. 1996). The synthetized insert contained 50 bp overlapping regions in both 5′ and 3′ ends to the vector to allow cloning using the Gibson assembly method (Gibson et al. 2009). The plasmids were transformed into *E. coli* BL21(DE3) strain for protein production. The *Cc xylC* gene was ordered as two different plasmid constructs with an eight amino acid long Strep-tag II (Trp-Ser-His-Pro-Gln-Phe-Glu-Lys) either at the N-terminus or at the C-terminus. A Site-directed Mutagenesis Kit (New England Biolabs, USA) was later used to remove the C-terminal tag from the *Cc xylC* gene, since it did not work in the purification procedure. The *Ec* y*agE* gene was tagged with a Strep-tag II at the N-terminus. A six amino acid long His-tag was added to the N-terminus for the *Ec aldA, Ab araE and Ec yqhD* genes, or to the C-terminus for the *Ec fucO* gene. All DNA constructs were verified by sequencing to confirm that no changes in the nucleotide sequence had occurred. Sequencing was done by Source Bioscience Sequencing, UK. The *Acinetobacter baylyii* ADP1 α-ketoglutarate semialdehyde dehydrogenase gene (GI: 81613403, NCBI) with an N-terminal His_6_-tag cloned into pET22 was a kind gift from Dr. Alain Perret, France.

Five genes encoding dehydratases from the enolase superfamily with putative activity on d-xylonate, were obtained as synthetic genes, codon optimized for *E. coli* and inserted into the pBAT4 vector (GenScript, China). The resulting plasmids contained the genes for the putative d-xylonate dehydratases from *Haloarcula marismortui* (*Hm* XylD, GI: 55230170, NCBI), and *Haloferax volcanii* (*Hv* XylD, GI: 292493977, NCBI), the putative d-gluconate dehydratase SEN1436 from *Salmonella enterica* (*Se* GluDHT, GI: 667467043, NCBI) the mandelate racemase/muconate lactonizing enzyme-like protein from *Rubrobacter xylanophilus* (*Rx* MR/MLE, GI: 123368307, NCBI), and the d-galactonate dehydratase family member RspA from Pantoea ananatis (*Pa* GalDHT, UniProt: D4GJ14). The dehydratases from *H. marismortui, H. volcanii*, and *S. enterica* were tagged with an N-terminal Strep-tag II, and a His_6_-tag was added to the C-terminal or N-terminal for the enzymes from *R. xylanophilus* and *P. ananatis*, respectively.

### Expression and purification of the pathway enzymes

The N-terminal Strep-tagged *Cc xylB* gene with a TEV site encoding the *Caulobacter crescentus* d-xylose dehydrogenase (*Cc* XylB) was expressed in *S. cerevisiae* under the PGK promoter, and *Cc* XylB was purified from the yeast cell extract in a single step using Strep-Tactin Sepharose. The buffer of the purified enzyme was changed to 50 mM phosphate buffer pH, 7 using PD-10 columns (GE Healthcare).

For purification of *C. crescentus* d-xylonolactonase (*Cc* XylC), *E. coli* BL21(DE3) cells containing the *Cc xylC* gene in the pBAT4-XylC plasmid were grown in Luria Broth medium (Bertani 1951) supplemented with 100 μg ml^−1^ ampicillin, at + 37 °C, 225 rpm to an OD_600_ 0.6–0.8. After the addition of 1 mM isopropyl-β-d-thiogalactopyranoside (IPTG) to induce expression of the gene of interest, the culture was further grown at + 30 °C, 225 rpm overnight. The cells were harvested by centrifugation at 4000×*g* for 20 min at 4 °C and suspended in ice-cold lysis buffer (50 mM Tris–Cl buffer, pH 7.5, containing 1 mM DTT, 1× protease inhibitor cocktail (Complete EDTA-free, Roche), DNAse I, RNase A, and lysozyme (Sigma-Aldrich)), and lysed by sonication. The cell-free sample was diluted ten times with 50 mM Tric-Cl, pH 7.5 and loaded onto a 20 ml DEAE FF 16/10 ion-exchange column (GE Healthcare) equilibrated with 50 mM Tris–Cl buffer, pH 7.5. After washing with 25 column volumes (CV) of equilibration buffer, the sample was eluted with a stepwise NaCl gradient (0–120 mM NaCl for 15 CV followed by 120–200 mM NaCl for 5 CV). The fractions were analyzed for *Cc* XylC content by SDS-PAGE analysis (10% Criterion SF gel, BioRad), and the fractions containing *Cc* XylC were pooled, concentrated and the buffer was changed to 50 mM Tris–Cl, pH 7.5 by Vivaspin 20 centrifugal concentrator (MWCO 10,000 Da, Sartorius AG, Goettingen, Germany).

The expression and purification of *C. crescentus* d-xylonate dehydratase (*Cc* XylD) was performed as described previously (Andberg et al. 2016). Briefly, the N-terminal Strep-tagged *Cc xylD* gene was expressed in *E. coli* BL21(DE3) cells at + 30 °C overnight. The enzyme was purified from the bacterial cell extract using a Strep-Tactin affinity column.

The *E. coli* 2-dehydro-3-deoxy-d-pentonate aldolase encoding gene (*Ec yagE*) was expressed in *E. coli* BL21(DE3) cells at + 30 °C overnight using enzymatic glucose release medium EnPresso B (Bio Silta Ltd). The cells were harvested by centrifugation 1800×*g* for 15 min at + 4 °C and suspended in ice-cold lysis buffer (100 mM Tris–Cl buffer and 150 mM NaCl, pH 8, supplemented with protease inhibitors, lysozyme, DNase I, and RNase A, and lysed by sonication. The sample was centrifuged at 24,000×*g* for 45 min at + 4 °C where after the clear cell extract was loaded on a 5 ml Strep Tactin column equilibrated with 100 mM Tris–Cl buffer and 150 mM NaCl, pH 8. The *Ec* YagE enzyme was eluted with 2.5 mM desthiobiotin and the fractions were analysed by SDS-PAGE. The fractions containing *Ec* YagE were pooled and concentrated on Vivaspin 20 (10000 MWCO) and the buffer was changed to 50 mM Tris–Cl, pH 8 using a PD-10 column (GE Healthcare Life Sciences).

The *E. coli* aldehyde dehydrogenase (*Ec aldA*) gene was expressed in *E. coli* BL21(DE3) in the pBAT4-AldA_N-6×His plasmid in LB medium supplemented with ampicillin at + 30 °C, 150 rpm overnight. Cells were harvested by centrifugation at 4000×*g* for 15 min at 4 °C and suspended in ice-cold lysis buffer (50 mM Tris–HCl pH 8, 300 mM NaCl buffer) and disrupted with two passes through a French press at 10,000 psi. The resulting cell lysate was incubated for 30 min in the presence of protease inhibitors, DNase I, RNase A and lysozyme after which the insoluble fraction was separated by centrifugation at 37,000×*g* for 20 min at 4 °C. For the purification of *E. coli* aldehyde dehydrogenase (*Ec* AldA), 20 ml of the cell-free extract was loaded onto a 5 ml HiTrap Chelating HP column (GE Healthcare) charged with NiSO_4_ and equilibrated with 50 mM Tris–HCl, pH 8, 300 mM NaCl. After the column was washed with 50 ml equilibration buffer, the bound fraction was eluted with a gradient (10 column volumes) from 0 to 500 mM imidazole. One millilitre fractions were collected during elution, and protein purity in the fractions was analysed with SDS-PAGE. The fraction containing *Ec* AldA were pooled and the buffer was changed to 50 mM Tris–HCl buffer, pH 7.5, 100 mM NaCl by gel filtration using an EconoPac 10 DG desalting column (Bio-Rad).

The N-terminally His-tagged *Acinetobacter baylii* ADP1 α-ketoglutarate semialdehyde dehydrogenase (*Ab* α-KGSA DH) was expressed and purified as described in (Aghaie et al. 2008). Briefly, the *E. coli* BL21/DE3 cells were grown in Terrific Broth medium containing 0.5 M sorbitol, 5 mM betaine, and 100 µg ml^−1^ carbenicillin at 37 °C until reaching an *A*_600_ of 2. After addition of 1 mM IPTG the culture was further grown at + 20 °C, 225 rpm overnight. The enzyme was purified from the cell-free extract in a single step using Ni–NTA chromatography and the enzyme was stored in 50 mM Tris–Cl buffer, pH 8, 50 mM NaCl, 10% glycerol and 1 mM DTT. The protein concentration of *Ab* α-KGSA DH was determined with the Bio Rad DC kit, using BSA as standard.

The genes corresponding to *Azospirillum brasilense* α-ketoglutarate semialdehyde dehydrogenase (*Ab araE*), *E. coli* 1,2-propanediol oxidoreductase (*Ec fucO*), and *E. coli* 1,3-propanediol oxidoreductase (*Ec yqhD*) were expressed in *E. coli* BL21(DE3) in the corresponding pBAT4-AraE_HisN, pBAT4-FucO_HisC, or pBAT4-YqhD_HisN plasmids. The cultivations were done in LB medium supplemented with ampicillin (225 rmp, overnight) at + 28 °C, + 22 °C, or at + 22 °C for *Ab* AraE, *Ec* FucO or *Ec* YqhD, respectively. The cells were harvested by centrifugation suspended in ice-cold lysis buffer (40 mM sodium phosphate buffer and 100 mM NaCl, pH 8 containing protease inhibitors, DNAse I, RNase A, and lysozyme, and disrupted by sonication. The samples were centrifuged at 12,000×*g* for 10 min at + 4 °C, where after the clear cell extract was adjusted to the binding buffer (20 mM sodium phosphate, 20 mM imidazole and 500 mM NaCl, pH 7.4) and loaded on a 5 ml HiTrap Chelating Sepharose column charged with Ni^2+^. The enzymes were eluted with 20 mM sodium phosphate, 200 mM imidazole and 500 mM NaCl, pH 7.4) and the fractions were analysed by SDS-PAGE. The fractions containing the enzymes of interest were pooled, concentrated, and the buffer was changed to 50 mM Tris–Cl, pH 7.5 using PD-10 columns for *Ab* AraE, or to 40 mM sodium phosphate buffer pH 7.4 using Econo-Pac column for *Ec* FucO and *Ec* YqhD.

### Enzyme activity measurements

The lactonase activity of *Cc* XylC was measured using d-xylono-1,4-lactone made fresh daily as the substrate. A circular dichroism (CD) -based assay for lactonase was performed in 10 mM Tris–HCl buffer, pH 6–8, using 1 mM lactone, with varying amounts of divalent metal ions and an aliquot of enzyme. CD spectra were recorded on a Chirascan CD spectrometer (AppliedPhotophysics, UK) equipped with a Peltier thermally controlled cuvette holder. Spectra were recorded using two scans, a bandwidth of 1 nm and a wavelength step of 0.5 nm, and the values were corrected for buffer contribution. The cuvette used for all measurements was a 1 mm and the temperature was set to 25 °C in all measurements.

The activity of *Cc* XylC was also followed by ^1^H-NMR. The reactions were carried out in 600 µl of 50 mM Na-phosphate buffer, pH 6.8 containing 10% of D_2_O (Aldrich) using 2 mM xylonolactone with 100 µM metal ions or EDTA. After recording a zero spectrum, 10 µg of *Cc* XylC was added and the reactions were followed at 22 °C by recording a series of 2 min ^1^H-NMR spectra. All NMR spectra were recorded on a 600 MHz Bruker Avance III NMR spectrometer equipped with QCI (H1/C13/N15/P31) cryoprobe and SampleJet sample changer. A modified version of SampleJet firmware allowed monitoring reactions in parallel while using the preheating block as an incubator. The water signal was suppressed by the so called 1D NOESY presaturation using Bruker’s pulse program noesygppr1d.

*Ec* AldA, *Ab* AraE and *Ab* α-KGSA DH dehydrogenases are NAD^+^- or NADP^+^-dependent enzymes whose activities were measured by following the increase in absorbance at 340 nm. Assays were performed at 22 °C, in 50 mM Tris–HCl buffer, pH 7, using 1–5 mM glycolaldehyde, 1 mM NAD^+^ (for *Ec* AldA and *Ab* AraE) or 1 mM NADP^+^ (for *Ab* α-KGSA DH), 0.5 mM DTT (for *Ec* AldA), 10 mM MgCl_2_ (for *Ab* AraE and *Ab* α-KGSA DH) and an aliquot of enzyme. The *Ec* FucO and *Ec* YqhD oxidoreductase activities were measured at 340 nm by following the oxidation of NAD(P)H at 22 °C in 50 mM Na-phosphate buffer, pH 7.5 using 10 mM glycolaldehyde and 1 mM NADH (for *Ec* FucO) or 1 mM NADPH (for *Ec* YqhD) as substrates.

The rate of glycolate, lactate or ethylene glycol formation in the last step of the different pathways was measured by following the NAD(P)H formation or consumption depending on the final dehydrogenase/oxidoreductase in the respective pathway. Assays were performed in microtitre plate at 22 °C in 50 mM Tris–HCl buffer pH 7, using 1 mM d-xylonolactone or d-xylonate, 2 mM NAD(P)^+^ or NAD(P)H, 10 mM Mg^2+^ and 0.5 mM DTT. The details of this method used to monitor the overall pathway are explained in more detail in the figure legends.

The production of glycolate starting from d-xylose utilising a 5-enzyme pathway was followed through an enzymatic assay using HAO1, which converts glycolate to glyoxylate and produced H_2_O_2_, which can be detected using HRP and Amplex red. The assay was carried out in a two-phase process, where the first phase contained 50 mM Tris–HCl pH 7.0, 1 mM d-xylose, 10 mM MgCl_2_, 2 mM NAD^+^, 2 µg *Cc* XylB, 8 µg *Cc* XylC, 4 µg *Cc* XylD, 4 µg *Ec* YagE and 4 µg *Ec* AldA (in a total volume of 200 µl). In this experiment, the concentration of the single enzyme in each 5 enzymatic step was varied keeping all others as in the basic reaction presented above. After this first 30 min reaction, the reaction was stopped by using 10.000 kDa cut-off Vivaspin centrifugal concentrator to separate the enzymes from the reaction product. The amount of glycolate produced in the first reaction was then measured after this separation step by using 50 µM Amplex Red, 0.4 µg HRP and 1 ug HAO1. This reaction was monitored at 560 nm for 1 h.

### Protein analysis

The purity of the enzymes was checked on SDS-PAGE gels using 4–20% or 10%, Criterion™ TGX Stain-Free™ Protein Gels (Bio-Rad, USA). For Western blot analysis the antibody used for proteins containing the Strep-tag II was Strep-Tactin^®^ conjugated to alkaline phosphatase (IBA, Germany), for proteins containing a His_6_ -tag an Anti-His tag Mouse Monoclonal (IgG2b) antibody was used (Trend Pharma & Tech Inc., Canada).

The protein concentrations for the purified enzymes were calculated from A_280_ using a theoretical extinction coefficient calculated by ProtParam (http://web.expasy.org/protparam/). The extinction coefficient for each enzyme is shown in parenthesis: *Cc* XylB (ε = 43,680 M^−1^ cm^−1^), *Cc* XylC (ε = 50,670 M^−1^ cm^−1^), *Cc* XylD (ε = 70,360 M^−1^ cm^−1^), *Ec* YagE (ε = 27,055 M^−1^ cm^−1^) *Ec* AldA (ε = 59,485 M^−1^ cm^−1^), *Ab* AraE (ε = 43,430 M^−1^ cm^−1^), *Ec* FucO (ε = 37,150 M^−1^ cm^−1^), and *Ec* YqhD (ε = 42,400 M^−1^ cm^−1^).

In order to determine the protein oligomeric state of *Cc* XylC, analytical gel filtration was conducted at 25 °C using a ACQUITY UPLC Protein BEH SEC 125 Å column (Waters Corporation) at a flowrate of 0.3 ml min^−1^. *Cc* XylC (0.6 μg protein) was injected on the column, pre-equilibrated in 100 mM sodium phosphate buffer pH 6.8. The absorbance of the eluate was monitored at 280 nm. Calibration of elution times was performed with the globular proteins ovalbumin (44.2 kDa, 0.3 ml min^−1^), ribonuclease A (13.7 kDa, 0.3 ml min^−1^), and uracil (112 Da, 0.3 ml min^−1^). The void volume of the column was determined with thyroglobulin (669 kDa, 0.1 ml min^−1^).

### Nucleotide sequence accession numbers

The nucleotide sequences reported in this study have been deposited in NCBI GenBank database under the accession numbers MH836586–MH836597.

## Results

**Fig. 2:**
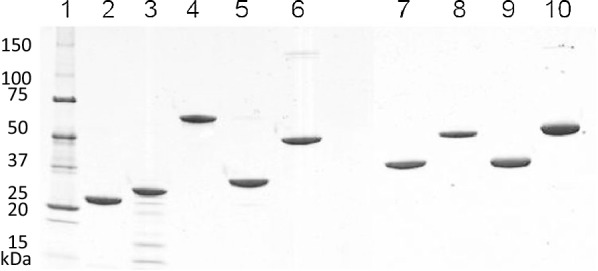
A SDS-PAGE showing the purification of the different pathway enzymes. A 10% SDS-PAGE gel was stained with Coomassie Blue. Lane 1 molecular weight marker proteins; lane 2, purified *Cc* XylB; lane 3, purified *Cc* XylC; lane 4, purified *Cc* XylD; lane 5, purified *Ec* YagE; lane 6, purified *Ec* AldA; lane 7, purified *Ec* FucO; lane 8, purified *Ab* αKGSA-DH; lane 9, purified *Ec* YqhD; lane 10, purified *Ab* AraE

For the in vitro metabolic pathway testing to convert d-xylose or d-xylonolactone to glycolate, l-lactate, or ethylene glycol (depicted in Fig. 1), altogether 14 different enzymes were expressed in heterologous host *E. coli* or *S. cerevisiae*. For protein purification, different tagging and purification strategies were used (Fig. 2 and Table 1).

### Characterisation of the *Cc* XylC lactonase

The first enzymatic step of these oxidative pathways is the oxidation of d-xylose to xylonolactone. We have earlier characterised several xylose dehydrogenases and shown that the xylose dehydrogenase from *C. crescentus* (*Cc* XylB) is an optimal enzyme in the metabolic pathways, as it is NAD^+^-dependent and specific for d-xylose, having no activity on hexose sugars (Toivari et al. 2012). The *Cc* XylB was thus the only enzyme considered for our in vitro pathway studies. The primary product of *Cc* XylB is xylonolactone, which is hydrolysed to linear d-xylonate form (Fig. 1). For this lactone ring opening reaction we were particularly interested to study and characterise the lactonase *Cc* XylC, which is found in *C. crescentus* in the same operon as the *Cc* XylB dehydrogenase and *Cc* XylD dehydratase enzymes (genes) (Fig. 1). The *Cc* XylC lactonase belongs to the human senescence marker protein 30, SMP30, family (EC 3.1.1.17; having 6-bladed beta-propeller fold). The enzyme was expressed in *E. coli*, purified and determined to be a monomeric protein, some oligomers with a higher molecular weight could also be observed (Additional file 1: Figure S1).

**Fig. 3:**
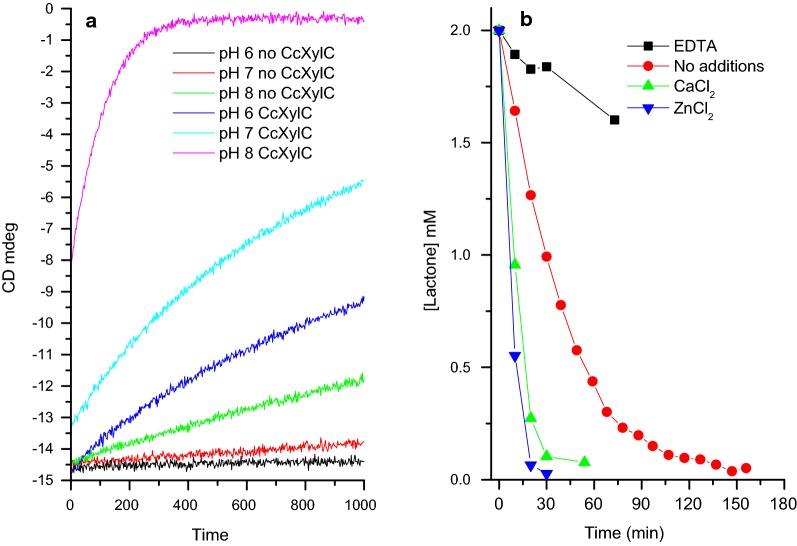
**a** pH dependency of the *Cc* XylC lactonase measured using circular dichroism. A circular dichroism (CD)—based assay for lactonase activity was performed in 10 mM Tris–HCl buffer, pH 6–8, using 1 mM lactone and 3.4 µg of enzyme (in case it was added to the reaction) in 300 µL buffer. **b** NMR based *Cc* XylC lactonase activity measurements. The reactions for NMR were carried out in 600 µl of 50 mM Na-phosphate buffer, pH 6.8 at 22 °C containing 10% of D_2_O using 2 mM xylonolactone with 100 µM metal ions or EDTA. After recording a zero spectrum 10 µg of *Cc* XylC was added

In CD spectroscopy d-xylonolactone gives a characteristic negative peak with a minimum at 220 nm, and disappearance of this lactone signal can be used to measure lactonase activity under different reaction conditions. In panel A of Fig. 3 the pH dependency of the lactone ring opening is shown both in the presence and absence of the *Cc* XylC lactonase. As expected, some non-enzymatic lactone ring opening reaction can be observed at pH 8. However, adding the *Cc* XylC lactonase results in a significant increase of the hydrolysis rate in the range from pH 6–8.

Lactonases are known to be metal binding enzymes and in particular, Zn^2+^ is reported to be found in the lactonases belonging to the SMP30 family (Chakraborti and Bahnson 2010). Currently it is not known whether the metal ion plays a role in the catalytic mechanism of lactonases. Using ^1^H-NMR to follow the lactonase reaction, we observed that although *Cc* XylC was able to open the xylonolactone without addition of any metal, the activity was clearly improved particularly in the presence of added Zn^2+^ and Ca^2+^ (Fig. 3b). Moreover, in the presence of EDTA to remove the metal(s), *Cc* XylC showed no activity, which demonstrates the importance of metal cations in the enzymatic lactone opening reaction.

### Screening for xylonate dehydratases

In the next step towards in vitro pathways we were screening xylonate dehydratases, which carry out the dehydration of the d-xylonate (Fig. 1). We have earlier experience that the dehydratase reaction might be a bottleneck in vivo due to the *Cc* XylD dehydratase enzyme, which is a tetrameric enzyme requiring a [2Fe–2S] cluster and Mg^2+^ ion for its activity (Andberg et al. 2016; Salusjärvi et al. 2017; Rahman et al. 2018). Five enolase family dehydratases, potentially active on d-xylonate were chosen from literature or databases, and expressed in *E. coli* for testing in the pathway (Table 1). SDS-PAGE analysis of whole cell extracts showed that all selected enzymes were possible to express in *E. coli*. The *Haloferax volcanii Hv* XylD and *Haloarcula marismortui Hm* XylD dehydratases were found only in the insoluble cell fraction which was the probable reason for not detecting any activity on d-xylonate. Neither was any d-xylonate activity detected for *Pa* GalDHT in cell extract although expressed as a soluble enzyme. The *Salmonella enterocolitica Se* GluDHT was shown to be precipitate and therefore no activity determinations could be performed. The purified *Rubrobacter xylanophilus Rx* MR/MLE catalysed dehydration of d-glucuronate, but not d-xylonate. Of the dehydratases tested in this work, the ILVD/EDD family *Cc* XylD dehydratase was found to be the best and was used in all in vitro pathway studies.

### Constructing in vitro pathways

**Fig. 4:**
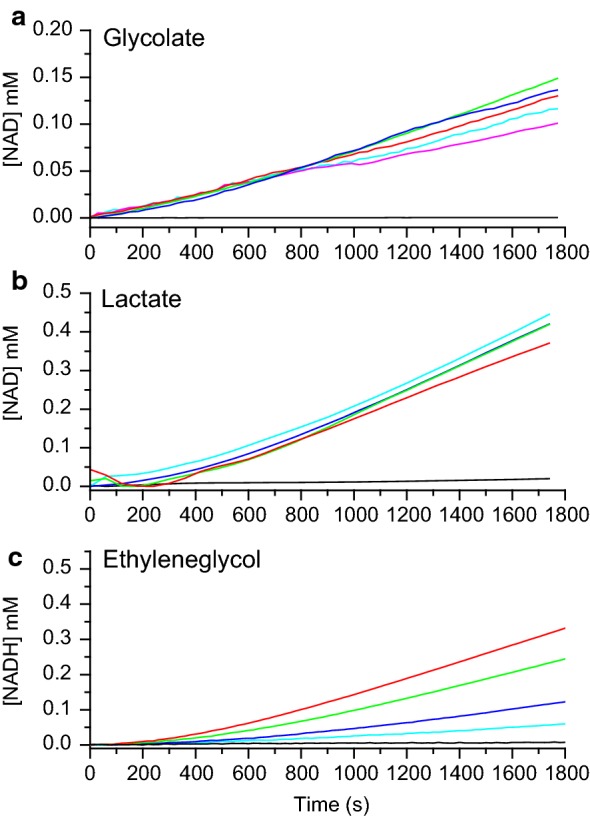
Evaluation of in vitro enzyme pathways for production of glycolate (**a**), lactate (**b**), and ethyleneglycol (**c**) (see also Fig. 1). In each case, the substrate for the pathway was xylonolactone and the pathway was composed of 4 enzymes, where the first three enzymes are common and the 4th is varied depending on the desired product. The production of glycolate was followed through generation of NADH, the production of lactate through generation of NADH, and the production of ethylene glycol was followed through generation of NAD^+^ by the final enzymatic step catalysed by *Ec* AldA (**a**), LDH (**b**), and *Ec* FucO (**c**) respectively. The reaction conducted at 22 °C contained 50 mM Tris HCl pH 7.0, 10 mM MgCl_2_, 0.5 mM DTT, 2 mM NAD^+^ or NADH, 1 mM xylonolactone, 4 µg *Cc* XylC, 2 µg *Cc* XylD, 2 µg *Ec* YagE. In **a** the amount of *Ec* AldA was varied 0 µg (black line), 2 µg (magenta line), 4 µg (cyan line), 8 µg (blue line), 16 µg (green line) and 24 µg (red line). In **b** the amount of LDH was varied 0 µg (black line), 0.1 µg (cyan line), 0.2 µg (blue line), 0.4 µg (green line), 0.8 µg (red line). In **c** the amount of *Ec* FucO was varied 0 µg (black line), 1 µg (cyan line), 2 µg (blue line), 4 µg (green line), 8 µg (red line)

We first evaluated the production of three different platform chemicals using in each case a 4-enzyme in vitro pathway, where the first three enzymes are common and the fourth is varied depending on which compound to produce. The efficiency of the pathway is in each case analysed using the final enzymatic step (oxidoreductase activity utilising NAD^+^/NADH) as a readout method. In Fig. 4 the overall activities of the pathways using xylonolactone as a substrate towards glycolate, lactate and ethylene glycol are shown using *Ec* AldA (Panel A), LDH (Panel B) and *Ec* FucO (Panel C) as the final oxidoreductase step respectively (see also Fig. 1). Clear activity was observed in all three pathways under the conditions stated in the figure legends, thus demonstrating that these pathways can be created in vitro.

**Fig. 5:**
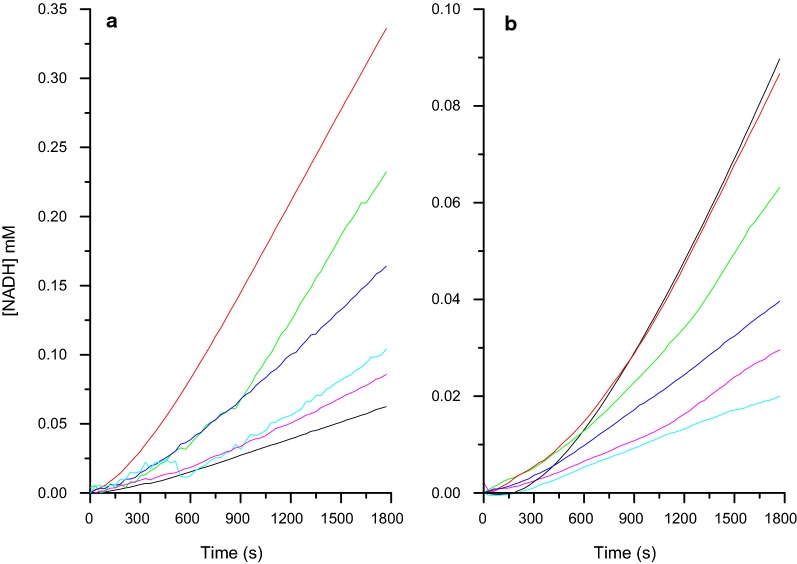
**a** Kinetic contribution of xylonolactonase to the in vitro pathway to glycolate using xylonolactone as a substrate. The effect of *Cc* XylC concentration to the glycolate production was measured at pH 7.0 at 22 °C and the production of glycolate was followed through generation of NADH from the enzymatic oxidation step from glycolaldehyde to glycolate. The assays were performed in microtiterplates containing 100 μl: 50 mM Tris HCl pH 7.0, 10 mM MgCl_2_, 0.5 mM DTT, 2 mM NAD, 1 mM xylonolactone, 2 µg *Cc* XylD, 2 µg *Ec* YagE, and 2 µg *Ec* AldA. The amount of *Cc* XylC in the reaction was varied 0 µg (black line), 2 µg (magenta line), 4 µg (cyan line), 8 µg (blue line), 16 µg (green line) and 24 µg (red line). **b** Effect of d-xylonolactone concentration on production of glycolate. The efficiency of the 4-enzyme pathway was followed through generation of NADH from the enzymatic reduction step from glycolaldehyde to glycolate. The assays were performed in microtiterplates containing 100 μl: 50 mM Tris HCl pH 7.0, 10 mM MgCl_2_, 0.5 mM DTT, 2 mM NAD, 0.5 mM xylonolactone (black line), 1 mM xylonolactone (red line), 2 mM xylonolactone (green line), 3 mM xylonolactone (blue line), 4 mM xylonolactone (cyan line) and 5 mM xylonolactone (magenta line), 4 µg *Cc* XylC, 2 µg *Cc* XylD, 2 µg *Ec* YagE, and 2 µg *Ec* AldA

In the next stage of the study, we focussed on the production of glycolate and looked in more detail into the conversion of d-xylose to glycolate and the contribution of the individual enzymes to this process. In the experiments presented in Fig. 5a we attempted to confirm the active role of the lactonase *Cc* XylC for the in vitro enzyme pathway to produce glycolate. The overall activity of the pathway was monitored by measuring the generation of NADH from the last enzymatic step catalysed by *Ec* AldA (which oxidises glycolaldehyde to glycolate) using xylonolactone as a substrate at pH 7.0, 22 °C. Our NMR analysis confirmed that at pH 7.0 xylonolactone ring is relatively stable, and freshly made xylonolactone solution is initially 95% in the lactone form (and 5% in the linear xylonic acid form) and after 9 h 75% in the lactone form (Additional file 2: Figure S2). In all experiments the xylonolactone was made fresh daily and used within 9 h. As seen from Fig. 5a, when xylonolactone was used as a substrate, little glycolate formation is observed before the addition of the lactonase, and only when the amount of *Cc* XylC in the reaction mixture was raised, an increase of glycolate formation is observed.

In order to assess the concentration influence of xylonolactone on the production of glycolate, xylonolactone concentrations were varied between 0 and 5 mM. Highest glycolate production was observed with 0.5–1 mM xylonolactone, and at concentrations above 1 mM, the glycolate production decreased in a concentration dependent manner, probably due to substrate inhibition (Fig. 5b). Thus 1 mM xylonolactone has been used in all pathway studies reported below.

**Fig. 6:**
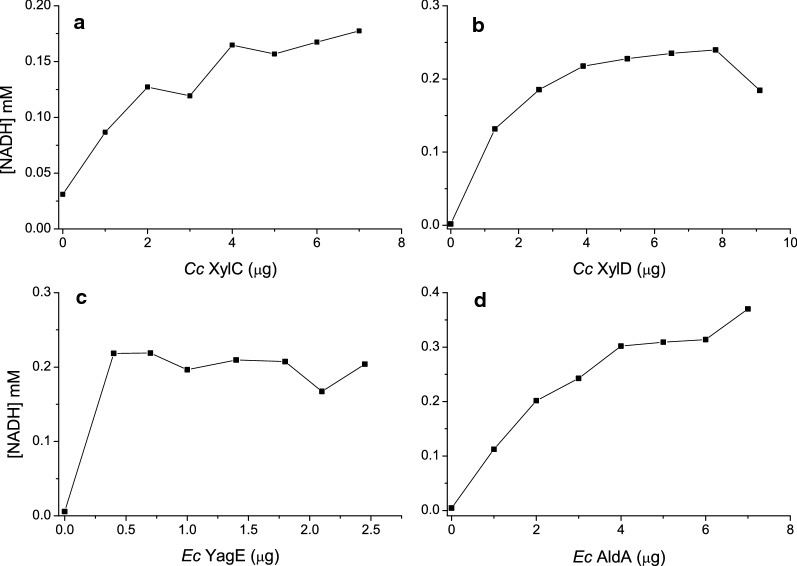
Optimisation of the in vitro pathway for glycolate production starting from d-xylonolactone. The assays were performed at 22 °C in microtiterplates containing 100 ul: 50 mM Tris HCl pH 7.0, 10 mM MgCl_2_, 0.5 mM DTT, 2 mM NAD, and 1 mM xylonolactone as the starting reaction conditions. The concentration of each enzyme in the pathway was varied in four separate experiments, where the concentrations of the other three enzyme were always fixed. The amount of glycolate produced was measured through NADH production (at 340 nm). The enzyme concentrations tested were: **a** *Cc* XylC (0–7 µg), *Cc* XylD (2 µg), *Ec* YagE (1.4 µg), *Ec* AldA (2 µg), **b** *Cc* XylC (6 µg), *Cc* XylD (0–9 µg), *Ec* YagE (1.4 µg), *Ec* AldA (2 µg), **c** *Cc* XylC (6 µg), *Cc* XylD (8 µg), *Ec* YagE (0–2.5 µg), *Ec* AldA (2 µg), **d** *Cc* XylC (6 µg), *Cc* XylD (7.8 µg), *Ec* YagE (0.7 µg), *Ec* AldA (0–7 µg)

Next, we monitored the rate of the glycolate production as a function of the other catalytic steps involved, using xylonolactone as the substrate (Fig. 6). In our 4-enzyme pathway, the concentration of each enzyme was varied in four separate experiments, keeping the concentration of the other three enzymes fixed. The amount of the varied enzyme corresponding to the highest glycolate production rate was used in the experiments for the following enzymatic steps. In the case of the first two, as well as the last reactions, a clear enzyme concentration dependency was observed. However, no real concentration dependence could be established for the third enzymatic step by the *Ec* YagE aldolase. The amount of *Ec* YagE aldolase needed in the pathway was clearly lower than any of the three other enzymes.

**Fig. 7:**
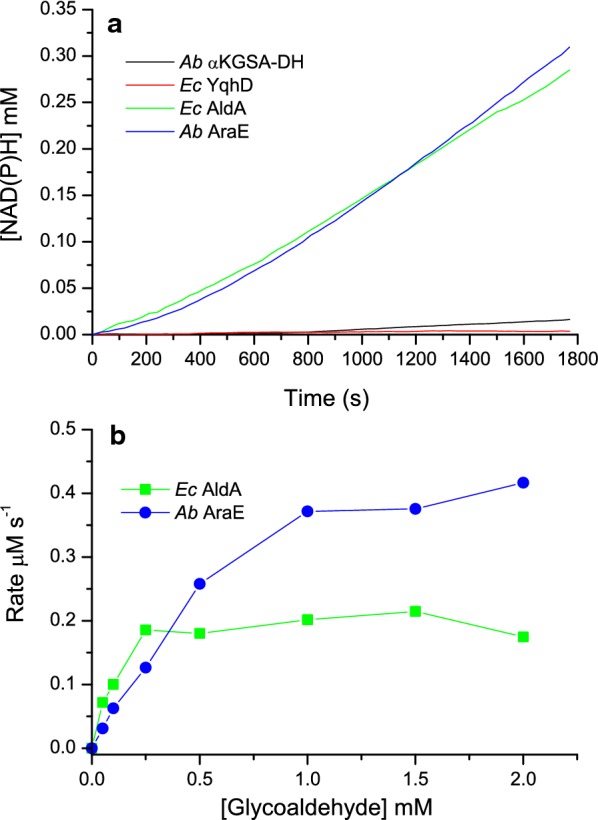
**a** Evaluating four alternative enzymes for the production of glycolic acid from glycolaldehyde. The production of glycolate was always followed through generation of NAD(P)H from the enzymatic oxidation step from glycolaldehyde to glycolic acid using 110 µg *Ab* αKGSA-DH (black line), 1.5 µg *Ab* AraE (blue line), 2 µg *Ec* AldA (green line) and 140 µg *Ec* YqhD (red line). The reaction mixture contained 50 mM Tris HCl pH 7.0, 10 mM MgCl_2_, 2 mM NAD or NAD(P), 1 mM DTT, 1 mM xylonolactone, 4 ug *Cc* XylC, 6 µg *Cc* XylD and 2 µg *Ec* YagE. **b** Glycolaldehyde concentration dependent steady-state kinetics comparison when using *Ec* AldA (circles) and *Ab* AraE (squares). The reaction contained 50 mM Tris HCl pH 7.0, 2 mM NAD, 1 mM DTT, glycolaldehyde 0–2 mM, 2 µg *Ec* AldA or 1.5 µg *Ab* AraE

After this we focussed our study to the final enzymatic step towards glycolate by testing three alternative aldehyde dehydrogenases as comparison to the originally used *Ec* AldA (Fig. 7a). Two of the tested enzymes, *Ec* YqhD and *Ab* α-KGSA-DH, showed no and very little activity compared to *Ec* AldA. *Ec* YqhD is known to catalyse the reduction of glycolaldehyde and it is therefore unlikely that it can also work as an oxidase for the same substrate. *Ab* AraE, on the other hand, performed similarly to *Ec* AldA when measured as part of the in vitro pathway. In Fig. 7b we compare the glycolaldehyde dependent activity of *Ec* AldA and *Ab* AraE. At low glycolaldehyde concentration (below 0.3 mM) the activity of both enzymes is similar, but at higher concentrations (above 0.5 mM), *Ab* AraE is more active. This seems to be due to an inhibitory effect observed in the case of *Ec* AldA, i.e. at high substrate concentrations we see an inhibition of the activity that is likely to be caused by either the substrate (glycolaldehyde) or the product (glycolate) of the reaction. This inhibition has been previously reported (Baldomà and Aguilar 1987).

**Fig. 8:**
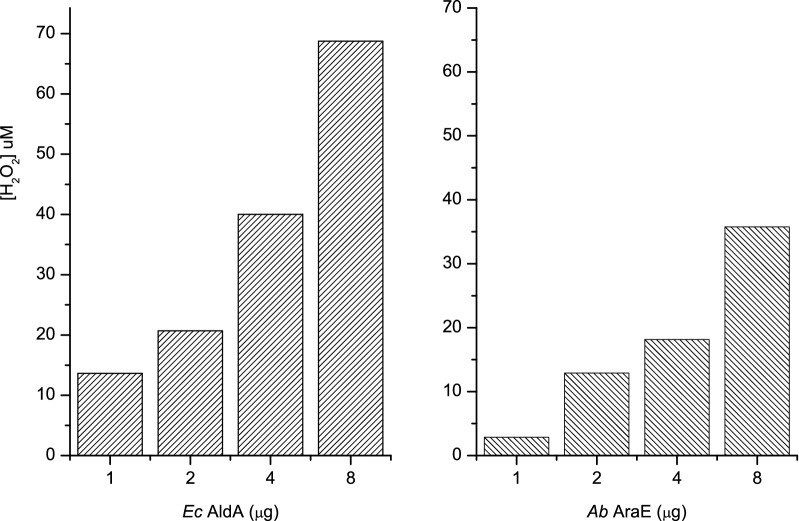
Evaluation of the final dehydrogenase of the in vitro enzymatic pathway from d-xylose to glycolic acid. The in vitro pathway is composed of 5 enzymatic steps (*Cc* XylB, *Cc* XylC, *Cc* XylD, *Ec* YagE and *Ec* AldA or *Ab* AraE). The production of glycolate was followed through an enzymatic assay using HAO1, which converts glycolate to glyoxylate and produced H_2_O_2_, which can be detected using HRP and Amplex red. The assay was performed as a two-step process as described in “Materials and methods”. The two final dehydrogenases of the pathway *Ec* AldA and *Ab* AraE are compared using this method

Finally we studied the in vitro production of glycolate using the complete 5-enzyme pathway starting from d-xylose, and testing both *Ec* AldA and *Ab* AraE dehydrogenases for the final step from glycolaldehyde to glycolate. As these 5-enzyme pathways contain two NAD^+^-dependent dehydrogenases, the NADH formation cannot be any more used as a readout for the formation of glycolic acid. We used instead here an indicator enzyme called HAO1, which catalyses the O_2_ dependent oxidation of the pathway product, glycolate, to glyoxylate and H_2_O_2_. The amount of H_2_O_2_ produced can in turn be detected using HRP peroxidase and Amplex Red as the substrate, resulting in the formation of resorufin, which can be measured spectroscopically (Wang et al. 2016). The results of these in vitro pathway experiments are shown in Fig. 8. As can be seen an increase in the amount of both dehydrogenases, *Ec* AldA and *Ab* AraE, results in an increase in glycolate production. It furthermore indicates that *Ec AldA* might be more efficient in the final step, possibly due to slightly higher activity at low glycolaldehyde concentration as shown in Fig. 7b. On the whole, both *Ab* AraE and *Ec* AldA seem to be suitable candidates for glycolate production in vitro as was also predicted based on the experiments presented in Fig. 7.

## Discussion

In this article, we reconstitute an oxidative d-xylose pathway in vitro to produce glycolate, lactate or ethylene glycol, and study the individual enzymatic catalysts of the pathway. The pathway is particularly of interest because of the aforementioned biotechnological applications. We were able to purify all the activities needed to reconstitute the oxidative d-xylose (or Dahms) pathway in vitro to produce biotechnologically important products (Fig. 1). We chose to use pH 7.0 for the in vitro pathway studies reported here, as it is close to the physiological pH of yeast or bacteria (Zilberstein et al. 1984; van Eunen et al. 2010). The characterisation of the pathway was based on the detection of NAD(H) as a readout for the formation of the products of interest.

As explained above and reported earlier by us (Toivari et al. 2012), oxidation of d-xylose by the xylose dehydrogenase produces xylonolactone as the primary product. The xylonolactone ring can open spontaneously to linear d-xylonate, particularly at alkaline pH. On the other hand, lactonases are frequently found in nature and observed in oxidative sugar pathways of bacteria and archaea. Our earlier studies have shown that the *Cc* XylC lactonase may have a beneficial role in the xylonic acid production in yeasts, however, its exact role has been difficult to address as the export of the acids and cell viability affect to the interpretation of the in vivo metabolic pathway studies (Brouns et al. 2006; Toivari et al. 2012; Salusjärvi et al. 2017). The studies by Nygård et al. (2014) demonstrated that while *Cc* XylC facilitated rapid opening of the xylonolactone, an accumulation of both d-xylonolactone and d-xylonate occurred during the microbial d-xylonate production.

Lactonases are, in general, quite poorly characterized enzymes due to challenges in the activity measurements. Lactonase activity is often measured using a simple pH shift assay, which is monitored with pH indicators such as p-nitrophenol. This type of assay can, however, only be used in a very limited pH window due to the nature of the reaction. We were interested in determining the pH and metal dependency of the lactonase-catalysed reaction by *Cc* XylC and used CD spectroscopy and NMR to follow the reaction. These measurements confirmed the potential significance of lactonases in oxidative pentose sugar pathways at neutral pH. At higher pH values the spontaneous non-enzymatic hydrolysis reaction also contributes to the overall rate. We were additionally able to confirm the importance of metal cations, such as Zn^2+^ and Ca^2+^, in the enzymatic lactone opening reaction by the *Cc* XylC.

Our earlier studies showed that the third enzymatic step of the pathway, i.e. a dehydration reaction to convert d-xylonate to 2-keto-3-deoxy-xylonate, that is carried out by a xylonate dehydratase enzyme (*Cc* XylD) which requires a [2Fe–2S] cluster as the cofactor (Rahman et al. 2018), seems to be a bottleneck particularly under the in vivo conditions (Salusjärvi et al. 2017). Sugar acid dehydratases can be found in several enzyme families, including the ILVD/EDD family and enolase family. The enolase family enzymes require only a metal for their catalytic activity and are considered to be easier to express than the more challenging FeS cluster containing ILVD/EDD enzymes. Therefore, we wanted to test other, enolase family dehydratases that do not require a FeS complex and could carry out the dehydration step. However, none of the selected enzymes were found to be better candidates for the dehydration reaction, either due to challenges to express the enzymes in soluble form, or enzyme stability, or the enzymes preferred other sugar acid substrates than d-xylonate.

After the initial characterization of lactonase and dehydratases we showed that a cascade reactions towards glycolate, lactate and ethylene glycol can be built in vitro (Fig. 4) In the case of *Ec* FucO (i.e. ethylene glycol production), an increase of the enzyme concentration resulted in an increase in the production levels, whereas increasing the enzyme concentration of the final enzyme has less of an effect in the case of *Ec* AldA (i.e. glycolate production) and LDH (i.e. l-lactate production). This suggests that the activity of the *Ec* FucO to produce ethylene glycol is rate-limiting, while in the case of the glycolate and l-lactate production, already the lowest enzyme concentration used (of *Ec* AldA and LDH, respectively) was sufficient.

Out of the three tested end-products, the glycolic acid is the most versatile biochemical that can be used as such, converted to glycolic acid containing homo- or heteropolymers, or further converted to acrylic acid and related products. Our experiments show that the *Cc* XylC contributes to the overall rate of glycolate formation and verifies the role of lactonase in this type of pathways (Fig. 5). With no lactonase present, the spontaneous opening of xylonolactone limits the overall rate of this multi-enzyme catalytic process. Furthermore, we observed an inhibition at higher xylonolactone concentration. This might also have significance in vivo in case accumulation of the lactone occurs.

In further characterisation of this 4-enzyme pathway, the contribution of each of the four enzymes was assessed (Fig. 6). Here, a clear enzyme concentration dependency was observed for the lactonase, dehydratase and dehydrogenase reaction steps, on the other hand *Ec* YagE aldolase seemed not to be rate-liming. Concerning the last enzymatic step to convert glycolaldehyde to glycolic acid, four different aldehyde dehydrogenases were tested (Fig. 7). Two of these, *Ec* AldA and *Ab* AraE dehydrogenases, seemed promising, *Ec* AldA possibly being the best option (Fig. 8). In order to assess this whole 5-enzyme pathway to produce from d-xylose glycolate we needed to construct another type of simple detection system. Here, a commercially available HAO1 turned out to be a useful indicator enzyme to measure the overall activity of the pathway.

Overall, our experiments show that valuable data on the contribution of an individual d-xylose (or Dahms) pathway enzymes can be obtained through in vitro pathway studies, enabling the optimization of the enzyme mixture for maximum product formation. As shown, this methodology also enables the selection of good enzyme catalyst for a single step if several potential candidates are available. In addition, several other factors can be studied using in vitro pathways: contribution of enzymatic steps (i.e. lactonase reactions), inhibitory effects, incompatibility of individual enzymes, and the ability of enzymes to function at a pH value that might be suboptimal for some of the individual enzyme catalysts. To be able to develop in vitro pathways further, detection of the all the intermediates in a time-dependent manner will be required. This type of data will allow simulation and modelling of such complex systems and thereby enhance the understanding of the observed non-linear and complex overall kinetic behaviour. Furthermore, we anticipate that in future more biotechnologically important pathways will be reconstructed in vitro to the assist the design of novel in vivo metabolic networks.

## Additional files

**Additional file 1.** Analytical gel filtration.

**Additional file 2.** ^1^H-NMR analysis of D-xylono-1,4-lactone.
