# Supplementary material for: In vitro reconstitution and characterisation of the oxidative d-xylose pathway for production of organic acids and alcohols
Source: AMB Express. 2019 Apr 11;9:48. doi: 10.1186/s13568-019-0768-7 (PMC6458216; doi:10.1186/s13568-019-0768-7)

***In vitro* reconstitution and characterisation of the oxidative D-xylose pathway for production of organic acids and alcohols**

Harry Boer^*^, Martina Andberg, Robert Pylkkänen, Hannu Maaheimo, Anu Koivula

VTT Technical Research Centre of Finland Ltd., P.O. Box 1000, FI-02044 VTT, Finland

**Corresponding Author:**

*E-mail: [harry.boer@vtt.fi](mailto:harry.boer@vtt.fi), Telephone: +358-20-7225183, Telefax: +358-20-7227071

Keywords: Dahms pathway, *in vitro* enzyme pathway, glycolate, ethylene glycol, lactate, lactone, lactonase

**Additional file 1: Figure S1** Analytical gel filtration in order to estimate the protein oligomeric state of *Cc* XylC at 25°C. The absorbance of the eluate was monitored at 280 nm. The peak observed at 4.4 min with a calculated molecular weight of 29 kDa corresponds to the *Cc* XylC monomer. Around 3.4 min higher molecular weight oligomers are observed. This retention time corresponds to a molecular weight of approximately 150 kDa


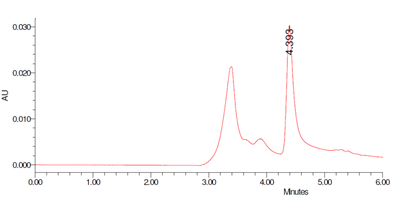

Supplement: Supplementary file 1 — Additional file 1. Analytical gel filtration. [file 13568_2019_768_MOESM1_ESM.docx]
