# Supplementary material for: In vitro reconstitution and characterisation of the oxidative d-xylose pathway for production of organic acids and alcohols
Source: AMB Express. 2019 Apr 11;9:48. doi: 10.1186/s13568-019-0768-7 (PMC6458216; doi:10.1186/s13568-019-0768-7)
Supplement: Supplementary file 2 — Additional file 2. 1H-NMR analysis of D-xylono-1,4-lactone. [file 13568_2019_768_MOESM2_ESM.docx]

***In vitro* reconstitution and characterisation of the oxidative D-xylose pathway for production of organic acids and alcohols**

Harry Boer^*^, Martina Andberg, Robert Pylkkänen, Hannu Maaheimo, Anu Koivula

VTT Technical Research Centre of Finland Ltd., P.O. Box 1000, FI-02044 VTT, Finland

**Corresponding Author:**

*E-mail: [harry.boer@vtt.fi](mailto:harry.boer@vtt.fi), Telephone: +358-20-7225183, Telefax: +358-20-7227071

Keywords: Dahms pathway, *in vitro* enzyme pathway, glycolate, ethylene glycol, lactate, lactone, lactonase

**Additional file 2: Figure S2** ^1^H-NMR analysis of D-xylono-1,4-lactone in 50 mM Na-phosphate buffer, pH 7.0, immediately after dissolving (green, 95% lactone) and after 9 h (red, 75% lactone) at 22°C. A small change in pH due to xylonic acid formation is obvious from the shift of the lactone signals during the 9 h. The blue spectrum is a reference spectrum of D-xylonic acid in the same buffer.
